# StudiCare mindfulness—study protocol of a randomized controlled trial evaluating an internet- and mobile-based intervention for college students with no and “on demand” guidance

**DOI:** 10.1186/s13063-020-04868-0

**Published:** 2020-11-26

**Authors:** Ann-Marie Küchler, Dana Schultchen, Olga Pollatos, Morten Moshagen, David D. Ebert, Harald Baumeister

**Affiliations:** 1 Department of Clinical Psychology and Psychotherapy, Institute for Psychology and Education, Ulm University, Albert-Einstein-Allee 47, 89081 Ulm, Germany; 2 Department of Clinical and Health Psychology, Ulm University, Ulm, Germany; 3 Department of Quantitative Methods in Psychology, Ulm University, Ulm, Germany; 4 Department of Clinical, Neuro- & Developmental Psychology, Vrije Universiteit Amsterdam, Amsterdam, Netherlands

**Keywords:** College students, University students, Mindfulness, Depression, Anxiety, Internet- and mobile-based interventions, E-health, Efficacy, Moderators and mediators, Guidance on demand

## Abstract

**Background:**

College is an exciting but also challenging time with an increased risk for mental health issues. Only a minority of the college students concerned get professional help, a problem that might be improvable by internet- and mobile-based interventions (IMIs). However, adherence of IMIs is a concern. While guidance might be a solution, it is resource-intensive, derailing potential implementation on population level. The first aim of this trial is to evaluate the efficacy of the IMI *StudiCare Mindfulness* (StudiCare-M) for college students with “on demand” and no guidance. The second aim is to examine potential moderators and mediators, contributing to the questions of “how” and “for whom” such interventions work.

**Methods:**

In this three-armed randomized controlled trial, both an unguided and “guidance on demand” (GoD) condition of StudiCare-M are compared to a waitlist control group. StudiCare-M is based on principles of acceptance and commitment therapy and stress management and consists of 7 modules plus two booster sessions. Participants in the GoD condition may ask their e-coach for support whenever needed. A total of 387 college students with moderate to low mindfulness are recruited at 15+ cooperating universities in Germany, Austria, and Switzerland via circular emails. Assessments take place before as well as 1, 2, and 6 months after randomization. The primary outcome is mindfulness. Secondary outcomes include stress, depression, anxiety, interoception, presenteeism, wellbeing, intervention satisfaction, adherence, and potential side effects. Among examined moderators and mediators are sociodemographic variables, pre-treatment symptomatology, treatment expectancy, self-efficacy, cognitive fusion, emotion regulation, and alexithymia. All data will be analyzed according to intention-to-treat (ITT) principles.

**Discussion:**

Providing effective interventions to help college students become more resilient can make a valuable contribution to the health and functionality of future society. If effective under the condition of minimal or no guidance, StudiCare-M offers a low-threshold potentially resource-efficient possibility to enhance college student mental health on a population level. Moderation- and mediation analyses will deliver further insights for optimization of target groups and intervention content.

**Trial registration:**

WHO International Clinical Trials Registry Platform via the German Clinical Studies Trial Register DRKS00014774. Registered on 18 May 2018.

## Introduction

The college years are an exhilarating time for students, but also involve multiple stressors such as moving away from home, finding a new peer group, and dealing with academic pressure [1]. As a consequence, this period of life comes with an increased risk for the development of mental health problems [2, 3]. Most mental disorders have their onset by the age of 24, when many young people are attending college [4]. In a recent WHO survey across 21 different countries (*n* = 1527), almost 25% of interviewed college students met DSM-IV criteria for at least one mental disorder in the last 12 months, with anxiety and mood disorders being the most prevalent [1]. Similar or even higher prevalence rates have been reported in many studies [5–8]. Mental health disorders among college students are related to various negative outcomes, such as poorer academic performance [9, 10] and higher college dropout rates [4, 11]. Unfortunately, less than 30% of the affected students receive minimally adequate treatment [1, 5, 12]. Because early treatment can prevent the onset of mental disorders [13] and thereby contributes to academic success, easily accessible low-threshold prevention and early intervention strategies are needed to help students develop effective coping strategies [14].

Interventions promoting student wellbeing, such as mindfulness trainings, offer a promising way to increase college student mental health. Such interventions train general coping and stress management skills [15] and are arguably less stigma-prone than disorder-specific support offers [16]. In line with assumptions of positive psychology, mindfulness trainings emphasize personal growth and strengthening of resilience rather than the removal of symptoms or disorders [17]. For example, mindfulness has been shown to be associated with enhanced emotion-regulation and therefore represents a protective factor in the face of internal and external stressors [18]. Lately, technology-based mindfulness interventions have been subject of a growing number of studies. Whereas internet and mobile-based interventions (IMIs) have shown to be equally effective as face-to-face treatments for numerous mental health problems [19], they also have several advantages over these traditional formats such as flexibility regarding time and place [20] and anonymous participation [21]. As these interventions can be designed in a cost-effective way, they have the potential to provide a large number of students with effective prevention and treatment options [22]. Finally, IMIs are especially suitable for this population because college students commonly seek health information online and show high acceptance of online mental health interventions [14, 23, 24].

The efficacy of mindfulness-based IMIs has been demonstrated in a number of studies and systematic reviews. In their meta-analysis, Spijkerman, Pots, and Bohlmeijer [25] examined 15 randomized controlled trials investigating adults with various mental disorders as well as healthy populations. Mindfulness interventions were compared to passive control groups (*N* = 10), active control groups (*N* = 5), or both (*N* = 2). The authors found small to medium effect sizes concerning the improvement of depression (*g* = 0.29, 95% CI 0.13–0.46), anxiety (*g* = 0.22, 95% CI 0.05–0.39), well-being (*g* = 0.23, 95% CI 0.09–0.38), and mindfulness (*g* = 0.32, 95% CI 0.23–0.42). The largest effect was shown for stress (*g* = .51, 95% CI 0.26–0.75). In another meta-analysis [26] of eight preventive mindfulness-based IMIs for non-clinical populations, similar effects were found compared to mostly (*N* = 7) passive control groups (*g* = 0.28–0.43, 95% CI 0.15–0.67) and were even larger at follow-up (*g* = 0.47–0.70, 95% CI 0.14–1.13). Even though these results are promising, there are still some questions that demand further examination.

To begin with, a majority of studies have used clinical populations, the general population, or employees. RCTs focusing on college student samples suggest comparable efficacy [27–31], but are often compromised by methodological limitations such as small sample sizes [29–31] or no long-term follow-up [27–29, 31]. Consequently, there is a need for large well-designed RCTs to confirm previous findings.

While unguided IMI formats can be effective, evidence suggests superior efficacy of guided IMI. In such guided IMIs, participants receive human support for working through the intervention, e.g., by an e-coach (health professional) giving them feedback and answering their questions. It has been proposed that the superior efficacy of guided IMIs might be due to increased intervention adherence [32]. Adherence is very relevant for mindfulness trainings, as mindfulness skills can only be developed with regular practice [18]. Indeed, unguided mindfulness IMIs have shown intervention dropout rates around 40–60% [29, 33, 34] and seem to be less efficacious then guided ones [25]. However, providing guidance also comes with increased intervention costs and therefore has implications for dissemination and scalability [25, 32]. These barriers might be overcome by using minimal guidance formats that combine the lower costs of unguided IMIs with the lower attrition rates of guided IMIs. One of these formats is called guidance on demand (GoD). In contrast to usual guidance formats, guidance by a therapist or e-coach will only take place when participants ask for it [32]. So far, only two trials examined GoD-IMIs, delivering cognitive behavioral therapy (CBT) to people with social phobia [35] and tinnitus [36]. Contrary to what one would expect, both studies did not find any significant differences in efficacy or adherence of GoD-IMIs compared to unguided IMIs. However, the social phobia trial [35] also found no differences between the GoD and a guided version nor the guided and an unguided version, which is somewhat surprising considering previous evidence for the superiority of guided IMIs [25]. Those results might be explainable by the fact that samples in both trials were highly burdened and therefore very motivated, irrespective of guidance [36]. In line with this, intervention dropout in the two studies was rather low and comparable in all conditions (20–30%). It remains to be examined whether these results can be generalized to mindfulness IMIs designed for non-clinical target-groups typically showing lower adherence.

Finally, the questions of how and for whom exactly mindfulness-based IMIs (as well as IMIs in general) work are yet to be answered [25, 37]. As large-scale RCTs provide an excellent framework for moderation and mediation analyses, they should routinely incorporate such analyses [38]. Therefore, in the current study, we will exploratively examine various potential moderating variables such as sociodemographic variables, pre-treatment symptomatology and treatment expectancy. From theory and current evidence, we also deduced a number of potential variables mediating the effect of mindfulness IMIs on mental health. Those include mindfulness itself [39], cognitive fusion as an aspect of psychological flexibility [39, 40], emotion regulation [41] and clarity about one’s internal experience (operationalized via alexithymia) [42]. Additionally, we will look at self-efficacy, as empowerment and self-management are assumed to be crucial factors for the efficacy of self-help interventions [43].

The current study is an extension of a previous RCT that investigated the efficacy and acceptance of *StudiCare Mindfulness* (StudiCare-M), a guided internet-based intervention to enhance mindfulness and wellbeing in college students [44]. We will examine whether an adapted unguided as well as a GoD version can be effective, as these would offer affordable options for long-term implementation into student health promotion programs. The specific research questions are:

1. Are the unguided and GoD versions of StudiCare-M effective in enhancing mindfulness in college students compared to a waitlist control group?
2. Are these two versions also effective concerning the secondary outcomes depression, anxiety, stress, presenteeism, well-being and interoceptive sensibility?
3. Are the unguided and GoD versions of StudiCare-M associated with side effects or adverse events?
4. Are there any differences between the unguided and GoD version of StudiCare-M concerning efficacy, adherence, satisfaction, side effects, or adverse events?
5. Which factors are associated with, moderate or mediate the effects of StudiCare-M?

## Methods

### Study design

**Fig. 1:**
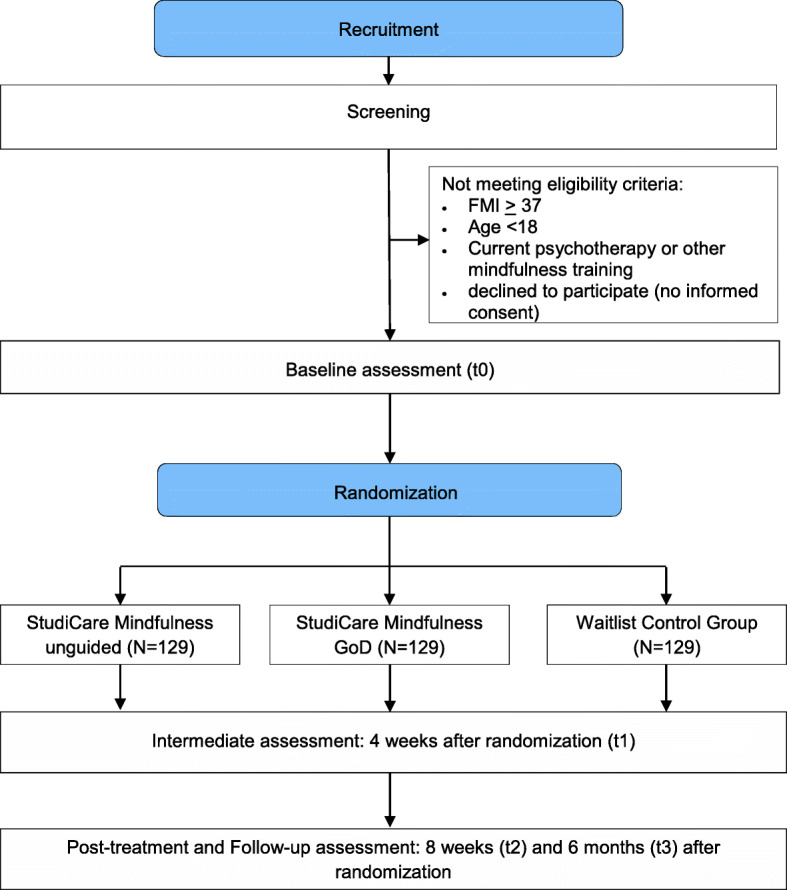
Flow diagram

This multicenter, three-armed randomized controlled trial of parallel design compares the efficacy of an unguided (UG) as well as a “guidance on demand” (GoD) version of the internet-based, preventive intervention *StudiCare Mindfulness* (StudiCare-M) to a waitlist control group (WL) receiving no intervention (superiority trial; see Fig. 1 for flowchart) within the framework of the StudiCare project funded by BARMER [45]. StudiCare dedicates itself to examining and promoting college students’ well-being offering a broad assortment of internet-based interventions for psychological and behavioral issues (e.g., procrastination, test anxiety, physical activity, depression, substance use, stress [20, 46–50]. It is embedded in the “World Mental Health Survey International College Student” project (WMH-ICS) [51] as well as the “Caring Universities” project [52].

The two versions of StudiCare-M are further compared to each other on an exploratory level to gain insights on potential differences in effectiveness, adherence, satisfaction side effects, and adverse events. Participants in all intervention arms are informed about and have access to treatment as usual. Use of other support options is monitored to control for potential confounding effects. The present study is conducted and will be reported according to the CONSORT 2010 Statement [53] and the guidelines for executing and reporting internet intervention research [54]. The study protocol follows recommendations of the SPIRIT 2013 Checklist for clinical trial protocols [55].

### Eligibility criteria

Participants providing written informed consent further need to meet the following inclusion criteria: (a) age 18 or above, (b) enrolled in university or college, (c) sufficient knowledge of German language (assessed via capability to proceed through enrollment and screening process), (d) internet access, (e) moderate to low mindfulness (Freiburg Mindfulness Inventory FMI ≤ 37); this cutoff was chosen as it represents the medium value of the FMI in subjects from the general population [56]. Participants are excluded from the study if they are undertaking psychotherapy or any kind of mindfulness intervention at the time of the screening.

### Setting/recruitment

Recruitment took place from May 2018 to April 2020. The main recruitment channel consists of circular e-mails sent out by more than 15 cooperating colleges in Germany, Austria, and Switzerland to all their students once every semester (for full list of cooperating colleges see StudiCare-Website [45]). Additionally, students are recruited via flyers and posters, social media, student unions, and student counseling. In the circular emails, students are informed about the StudiCare offers and are provided with a link to the StudiCare homepage [45], where they can obtain further information and register for the IMIs. Once registered, they receive an email with a link to the eligibility screening. Depending on which college participants attend, they are either allocated to a parallel local trial at Ulm University which combines StudiCare-M with on-site laboratory and psychophysiological measures [57] or to the present study (all other cooperating colleges). After successfully completing the screening, they receive an email with further information on the study as well as an informed consent form which they are required to send to the study team. When written consent is obtained and the pretest completed, another email with a link to the intervention is sent to participants randomized to one of the intervention groups. Participants are informed that StudiCare-M is not designed to replace psychotherapy, recommended to seek counseling/psychotherapy in case of distinctive mental health problems and provided with further treatment options and contact details. Participants of the WL will receive information on further study procedures as well as on alternative support options they can seek in case of deterioration of wellbeing during the waiting period.

### Randomization

After completing baseline assessment, participants are randomly allocated to one of three study groups (unguided, “guidance on demand,” waitlist control group) by an independent researcher not otherwise involved and therefore blinded to all processes of the study. Via an automated, online-based randomization program [58], permuted block randomization is performed with an allocation ratio of 1:1:1 and variable block sizes of 6, 9, and 12 (randomly arranged). As this is an open-label study, blinding of group allocation to participants and e-coaches will not be possible. Consequently, unblinding will not be necessary.

### Intervention

The intervention consists of seven weekly core modules of approximately 60 min. each. Additionally, two booster sessions are unlocked 4 and 12 weeks after completion of the seventh module to ensure sustainability of intervention effects. The main goal of the intervention is an increase in mindfulness and psychological flexibility. All modules contain information on stress, wellbeing, and mindfulness with a weekly alternating focus on different subjects such as interoception, dysfunctional thinking, or values and goals. Whereas these contents are provided via text, images, and interactive elements (such as quizzes or conditional content), the intervention also emphasizes the regular practice of mindfulness exercises like body scans and breathing meditations. Each module includes downloadable audio files as well as a mindfulness diary to be practiced in weekly homework assignments. At the beginning of each module, participants are encouraged to review their homework as well as their most and least mindful moments of the week. The content of the intervention is based on elements of acceptance and commitment theory (ACT) [59] as well as stress management principles [60]. ACT teaches acceptance, mindfulness, and value-based living and has found to be effective in the prevention of stress as well as the treatment of various psychological disorders [18]. The intervention was developed by the Department for Clinical Psychology and Psychotherapy, Ulm University. Its efficacy has already been demonstrated in a previous randomized controlled trial [44]. For the current RCT, the intervention was further refined and extended according to participants’ feedback. Table 1 summarizes the topics and contents of each module. The intervention is available to participants on the Minddistrict platform [61], a company specialized in the provision of internet-based health interventions. Participants are able to access the platform via their personal username and password on a 24/7 basis. All transferred data is secured based on ISO27001 and guidelines NEN7510.

**Table 1:** Intervention content

Module	Aims and content	Examples of exercises and assignments
1. Being in the here and now	Introducing the concept of mindfulness	Reviewing most and least mindful moments of the day; practicing body scan; taking mindful walk
2. Mindful body perception	Practicing awareness of body signals	Testing one’s heartbeat perception; practicing “heart meditation”; mindful eating and drinking
3. A new perspective on stress	Distancing oneself from stress-inducing thoughts	Identifying former ways of coping with stress; learning techniques to challenge automatic thoughts; meditation exercise
4. Developing beneficial thoughts	Getting to know alternative ways of thinking	Identifying one’s “stress patterns” and developing and internalizing beneficial thoughts; practicing breathing meditation
5. What makes your life valuable?	Identifying one’s values and pursuing one’s goals	Writing a speech for one’s 70th birthday; setting and pursuing goals with the SMART technique; meditation exercise
6. Being mindful towards yourself	Learning how to appreciatively accept one’s personality traits	Exercise to identify different personality traits and corresponding automatic reactions; learning to accept and appreciate all personality traits; loving kindness meditation
7. Training your body and senses	Exercising the ability to enjoy and getting acquainted with the practice of yoga	Mindful chocolate eating exercise; mindful yoga exercises
Booster 1 (4 weeks after completion of module 7)	Repeating module 1 to 3 and mindfulness exercises	Choosing favorite mindfulness exercises; setting goals for their implementation in the coming weeks
Booster 2 (12 weeks after completion of module 7)	Repeating modules 4 to 7 and ensuring long-term integration of mindfulness into daily life	Reviewing pursuit of goals in the last 2 months; identifying potential barriers and developing solutions

#### Guidance and promotion of adherence

Participants randomized to the GoD version of the intervention receive support by an e-coach whenever they desire to. E-Coaches are trained and supervised (by HB, AK) psychologists that give semi-standardized feedback on demand within 2 working days, following an e-coach manual. Whenever participants have questions or wish feedback on their module input, they can contact their personal e-coach via the platform’s message function. Each participant receives a welcoming message by their e-coach as well as a short introduction how to use GoD in the first module. Feedback content is specific to participants’ assignments and includes positive reinforcement, motivation, and encouragement. Actual usage of GoD by participants is documented. Participants in the unguided version of StudiCare-M do not receive any guidance. However, they receive short standardized automated feedback messages after completion of each module to reinforce and motivate them to continue the intervention. In both groups, participants are sent automated standardized e-mails by the Minddistrict platform if they have not logged in for more than 7 days. There will be no special criteria for discontinuing or modifying allocated interventions. However, all participants allocated to one of the two intervention versions will receive a document with descriptions and contact information of additional support offers (e.g., counseling, psychotherapy, emergency room), which they can refer to in case of symptom deterioration or need for more intensive care. Additionally, participants in the GoD version will be individually referred to additional support offers by their e-coaches in case of symptom deterioration. Post-trial care will be provided for both intervention arms in form of two booster sessions unlocking automatically after 1 and 3 months, with optional guidance by an e-coach for the GoD version. Finally, participants of both intervention arms will have unrestricted access to usual treatment options (such as psychotherapy or medication).

#### SMS coach

In the first module, participants of both IMI trial arms are offered the possibility to sign up for a text message coach. Once signed up, they receive standardized automated text messages every second day for the duration of the core intervention (8 weeks). Messages are designed to remind participants of their homework assignments, motivate them to integrate learned techniques into their daily life, and generally prompt them to be mindful during the day. Text message prompts have been shown to be useful in internet interventions regarding efficiency as well as adherence [62, 63].

#### Control condition

Participants in the waitlist control group have unrestricted access to usual treatment options (TAU). They receive an information leaflet informing them about alternative support options such as university counseling services, psychotherapy, or helplines as well as the encouragement to seek help in case of any deterioration of wellbeing. After t3 (6 months after randomization), WL participants receive the unguided version of the intervention.

### Assessments and outcomes

Assessment takes place before (t0; baseline) as well as 4 weeks (t1; intermediate), 8 weeks (t2; post-treatment), and 6 months (t3; follow-up) after randomization. t1 is an intermediate assessment of a subset of outcomes (see Table 2). All data will be self-reported and collected via the online survey platform “Unipark” [64]. Blinding of outcome assessment will therefore not be possible. To reduce assessment dropout, an email reminder strategy is employed. Participants’ phone numbers are collected on a voluntary basis to have the possibility of reminding them of filling out the surveys. Additionally, a raffle of 10 20-Euro “Amazon” coupons will take place among completers of the t3-survey at the end of the study.

**Table 2:** Outcomes and assessment points

Variables	Measurement	Screening	t0	t1	t2	t3
**Inclusion/exclusion criteria**						
Inclusion/exclusion criteria	SRQ	x				
Mindfulness	FMI	x				
**Primary outcome**						
Mindfulness	FMI				x	
**Secondary outcomes**						
Mindfulness	FMI		x	x		x
Depressive symptoms	PHQ-9		x	x	x	x
Anxiety	GAD-7		x	x	x	x
Stress	PSS-4		x	x	x	x
Well-being	WHO-5		x	x	x	x
Presenteeism	PSS		x		x	x
Interoceptive sensibility	BPQ		x		x	x
Self-efficacy	SES		x	x	x	x
Cognitive fusion	CFQ-D		x	x	x	x
Emotion regulation	ERQ		x	x	x	x
Alexithymia	TAS-20		x	x	x	x
Intervention adherence	Intervention dropout			x	x	x
Intervention satisfaction	CSQ-8				x*	
Side effects of intervention	INEP*				x	x
**Covariates**						
Demographic variables	SRQ		x			
Previous experience with mindfulness, use of additional treatment options	SRQ		x		x	x
Treatment expectation	CEQ		x			

*Note.* *CEQ* Client Expectancy Questionnaire, *CFQ-D* Cognitive Fusion Questionnaire, *CSQ-8* Client Satisfaction Questionnaire, *German Version* ZUF-8, *ERQ* Emotion Regulation Questionnaire, *FMI* Freiburg Mindfulness Inventory, *GAD-7* Generalized Anxiety Disorder Questionnaire, *INEP* Inventory for the Assessment of Negative Effects of Psychotherapy, *BPQ* Body Perception Questionnaire, Awareness Section, *PHQ-9* Patient Health Questionnaire, *PSS* Presenteeism Scale for Students, *PSS-4* Short Form Perceived Stress Scale, *SES* Self-Efficacy Scale, *SRQ* Self-Report Assessment Questionnaire, *TAS-20* Toronto-Alexithymia Scale, *WHO-5* World Health Organization Well-Being Index

*Intervention groups only (UG, GoD)

#### Primary outcome: mindfulness at post-treatment (t2)

The 14-item short scale of the Freiburg Mindfulness Inventory [56] is used to assess mindfulness. The FMI consists of a 4-point scale ranging from 1 = “rarely” to 4 = “almost always.” The short scale demonstrated sensitivity to change [56] and high internal consistency (*α* = 0.84) [65].

#### Secondary outcomes

##### Mindfulness at intermediate assessment (t1) and follow-up (t3)

Mindfulness is also measured after 4 weeks and 6 months.

##### Depressive symptoms

The depression module of the Patient Health Questionnaire [66] comprises of nine items that are rated on a 4-point scale (0 = “not at all” to 3 = “nearly every day”). The PHQ-9 is a widely used depression screening that has been shown to be a valid instrument [67] with good diagnostic properties and excellent internal consistency (*α* = 0.89). It has also been evaluated as an online version [68].

##### Anxiety

The 7-item Generalized Anxiety Disorder Questionnaire [69] is a screening instrument for generalized anxiety disorder and ranges from “not at all” (= 0) to “nearly every day” (= 3). The GAD-7 has been identified to be a reliable and valid measure of anxiety in the general population with a high internal consistency of Cronbach’s *α* = 0.89 [70].

##### Stress

The Short Form Perceived Stress Scale (PSS-4), derived from the Perceived Stress Scale [71], will be used to measure the participants’ perceived stress as the degree to which situations in one’s life are rated as stressful (scale ranging from 0 = never to 4 = very often). The psychometric properties of the PSS-4 have been found to be acceptable and reliable across cultures, with *α* = 0.77 [72].

##### Well-being

The well-established 5-item World Health Organization Well-Being Index (WHO-5) [73] is used to assess subjective psychological well-being. The scale ranges from “at no time” (= 0) to “all of the time” (= 5). Good psychometric properties of the WHO-5 as a screening tool for depression have been demonstrated among diverse clinical studies. Clinical validity has been identified as very high [74].

##### Presenteeism

As academic outcomes, presenteeism, loss of productivity, and absenteeism are assessed using a modified version [46] of the Presenteeism Scale for Students [75]. Presenteeism is measured by the subscale for work impairment (Work Impairment Scale; 10 items, scale 1–5, range 10–50). Productivity losses will be assessed by an adaption of the Presenteeism Scale for Students’ work output scale, investigating the current percentage to which participants were able to reach their usual academic productivity (visual analogue scale ranging from 0% = completely unproductive to 100% = full productivity). Additionally, hours of absenteeism are inquired. For the Work Impairment Scale, a Cronbach’s *α* of 0.90 as well as sufficient test-retest reliability and criterion-related validity could be demonstrated [75].

##### Interoceptive sensibility

Interoceptive sensibility (IS) is assessed by the awareness section of the Body Perception Questionnaire (BPQ) [76]. The section includes 54 items of subjective identifications of bodily signals on a 5-point scale, ranging from “never” (= 1) to “always” (= 5). High scores reflect poor IS. For the short form of the BPQ, categorical omega coefficients between 0.77 and 0.96 as well as high retest reliability were shown [77].

##### Subjective side effects and adverse events

The Inventory for the Assessment of Negative Effects of Psychotherapy (INEP) [78] assesses any changes experienced during or after the treatment in the social and/or work environment and whether they are attributed to the psychotherapeutic intervention. Four items are rated on a 7-point bipolar scale (− 3 = “worse,” + 3 = “better”); the others are rated on a 4-point scale (0 = “no agreement,” 3 = “full agreement”). In the present trial, an adapted 22-item version covering possible negative effects associated specifically with online-trainings (e.g., concerns about data protection) will be applied. The original scale has demonstrated high internal consistency with a Cronbach’s *α* of 0.86 [78].

##### Intervention satisfaction and adherence

The Client Satisfaction Questionnaire (CSQ-8) [79] is a validated 8-item instrument and is used in a version adapted for the evaluation of IMIs [80]. It comprises of eight items, each with a 4-point scale of specific response alternatives (e.g., 1 = “quite unsatisfied,” 4 = “very satisfied”). Good psychometric properties have been demonstrated including Cronbach’s *α* between .88 and .92 [81]. To operationalize intervention adherence, the number of completed modules is assessed. “Per protocol” adherence is operationalized by the percentage of participants that completed at least 5 of the 7 modules 8 weeks after randomization (t2). Additionally, quantitative and qualitative data are collected on participants’ satisfaction with various aspects of the intervention (e.g., number and length of modules, SMS-Coach, practicability in daily life) using self-constructed items (e.g., “Which elements did you find particularly helpful?”).

##### Use of GoD, subscription to SMS coach, and practice of mindfulness exercises

The number of times participants of the GoD condition contact their e-coach will be documented. We will also track whether participants of both intervention groups subscribe to the SMS-coach. Finally, we will assess the weekly time that participants spent practicing the mindfulness exercises introduced to them in the modules (retrospectively at t2).

#### Potential mediators

##### Self-efficacy

Perceived general self-efficacy is measured by the 10-item Self-Efficacy Scale [82] on a 4-point response scale from “1 = not at all true” to “4 = very true.” It was used in numerous research projects, where it demonstrated internal consistencies of Cronbach’s *α* = 0.75–0.91. It has also been proven reliable and valid in various field studies [83].

##### Cognitive fusion

In ACT, cognitive fusion is defined as the extent to which individuals identify with and are behaviorally regulated by their own thoughts and beliefs. Therefore, it is an important aspect of Psychological Inflexibility, which the intervention aims to reduce. It is assessed with the German version of the Cognitive Fusion Questionnaire [84]. Participants are asked to rate the seven items of the CFQ-D on a 7-point scale ranging from “1 = never true” to “7 = always true.” The CFQ-D has demonstrated good psychometric properties reflected in a Cronbach’s *α* of 0.95 as well as convergent validity with measures of physical and mental health [84].

##### Emotion regulation

The Emotion Regulation Questionnaire (ERQ) [85] is used to assess individual differences in habitual use of two emotion regulation strategies, reappraisal and suppression. Participants are required to indicate whether they agree with each statement on a 7-point scale ranging from 1 (= strongly disagree) to 7 (= strongly agree). The ERQ demonstrates good scale score reliability for the suppression (Cronbach’s *α* = 0.76) as well for the reappraisal factor (Cronbach’s *α* = 0.74) [86].

##### Alexithymia

The Toronto-Alexithymia Scale (TAS-20) [87, 88] is used to measure alexithymia. The questionnaire consists of 20 items rated on a 5-point scale (1 = strongly disagree; 5 = strongly agree) with total scores ranging from 20 to 100, reflecting three factor scales: “difficulties identifying feelings” (DIF), “difficulty describing feelings” (DDF), and “externally oriented thinking” (EOF). Higher scores on the different subscales indicate higher levels of alexithymia. The TAS-20 is a valid instrument with good internal consistency (Cronbach’s *α* = 0.85–0.86) and test-retest reliability [89].

#### Covariates

To investigate potential effect-modifying influences [90], several sociodemographic as well as other variables are assessed: age, gender, nationality, marital status, study course and number of semesters, previous experience with mindfulness, psychotherapy experience, and use of additional treatment options (such as psychological counseling or psychotherapy) as well as baseline symptomatology. To examine the influence of treatment expectations on outcomes, the Client Expectancy Questionnaire (CEQ) is used, which has demonstrated high internal consistency (*α* = 0.84–0.85) [91]. It consists of six items which are measured on a 9-point Likert Scale with higher scores representing positive expectations and credibility.

### Sample size estimation

Sample size calculations refer to detect an expected increased efficacy of StudiCare-M unguided and GoD compared to WL on the primary outcome mindfulness at t2 (post-treatment). In their meta-analysis, Spijkerman et al. (2015) found a small effect for unguided mindfulness IMIs (*g* = 0.22, 95% CI 0.10–0.34) and a significantly larger effect for guided IMIs (*g* = 0.43, 95% CI 0.30–0.56) [25]. The GoD version of our IMI provides the possibility to contact an e-coach at any time. Additionally, both the GoD and unguided versions contain various persuasive e-health technologies and that have been shown to increase efficacy and adherence (e.g., self-monitoring, goal setting, SMS prompts, automatic reminders) [63, 92]. Therefore, we assume an effect size comparable to the effects previously found for guided IMIs of *d* = 0.40. Originally, a power analysis based on a two-tailed t-test (calculated using G*Power [93]) resulted in a sample size of 133 participants per group. This was documented in the first version of the trial registration. However, in the meantime, a more precise power calculation was done by an independent biostatistician (MM). This calculation indicates that 129 participants per group are required to obtain a power of 1-beta = 90% based on *α* = .05 (taking into account clustering of participants by university and assuming an ICC = .02). Trial registration was updated accordingly. As data analysis will be based on intention-to-treat (ITT) principles, increasing sample size in order to compensate for drop-outs is not necessary.

Concerning an exploratory comparison between the UG and GoD version of StudiCare-M, the sample size is sufficient to detect a small effect of *d* = 0.20 as minimal clinically important effect difference with a power of 50% and a significance level of *α* = .05. This also corresponds to the range of effect sizes of *d* = 0.02–0.33 found for different outcome measures in the trials of Berger et al. [35] and Rheker et al. [36].

### Statistical analyses

All data analyses will be performed after completion of data collection. Interim analysis is not considered necessary as in the context of IMIs, there are no known dangers or harms that could make a trial stop necessary. Analyses will be performed on an intention-to-treat basis. Procedures of imputation will be chosen based on patterns and mechanisms of missingness (e.g., by using multiple imputation). Additionally, per protocol analyses (based on the data of participants that completed at least 5 of the 7 core modules) will be performed to examine the impact of drop-outs on study results. Significance level for all analyses will be defined as alpha = .05. A blinded analysis of primary and secondary outcomes will be conducted by an independent researcher not otherwise involved in the execution of the study (MM).

The primary outcome FMI at t2 will be analyzed by means of linear regression models. Baseline and group will be defined as predictors. Secondary outcomes will be analyzed accordingly. Standardized mean differences with 95% confidence intervals will be calculated post-treatment and follow-up to analyze between-group effect sizes. To obtain the number of participants achieving reliable improvement in mindfulness (FMI), participants will be coded as responders and nonresponders according to the Reliable Change Index (RCI) for t2 and t3 [94]. Additionally, we will investigate potential negative effects on individual level by calculating the number of participants that display a reliable deterioration from t0 to t2 and t3 also using the RCI [95, 96]. For an exploratory examination of associations between adherence, subscription to SMS-coach, actual use of GoD, time spent practicing mindfulness exercises, and the primary outcome FMI at t2 regression analyses will be conducted.

#### Moderator and mediator analyses

For the exploratory moderator analyses, regression models will be used. In a first step, each potential moderator defined in the “Covariates” section will be tested in a separate regression model. The primary outcome FMI at t2 will be set as dependent variable. Included predictors will be group, the respective moderator variable and the interaction of group and moderator. Subsequently, a comprehensive model of all identified moderators will be estimated.

Mediation analyses will be conducted according to the principles of time-lagged mediation by Cole and Maxwell [97]. This approach will enable the establishment of temporal precedence, an important requirement for the investigation of mechanisms of change [98]. Group will be set as independent variable, whereas the variables defined in the “Potentials mediators” section as well as mindfulness (FMI) will constitute the respective mediating variables. Depression (PHQ-9), stress (PSS-4), and anxiety (GAD-7) will be chosen as outcome variables, as they have been studied most frequently in previous mediation studies in the context of mindfulness interventions [39].

## Discussion

Psychological problems in college students are widespread and associated with poorer academic performance. Internet-based interventions (IMIs) for enhancing mindfulness are a promising way to increase student mental health but typically suffer from low adherence, compromising the effectiveness of such interventions. This trial will be the first to examine the effectiveness of a mindfulness IMI without and with guidance-on-demand, comparing both versions to a waitlist control group as well as to each other. Additionally, we will investigate potential moderators and mediators, an area that is still understudied [25, 99].

Despite best efforts, any trial comes with certain limitations that are described in the following.

A common problem with internet-based interventions is high dropout-rates concerning both intervention and assessments. For unguided mindfulness IMIs, intervention dropout rates of 40–60% have been reported suggesting that only about half of the participants complete such interventions [29, 31]. In order to overcome this problem, we will implement the following measures: an optional SMS-Coach prompting participants every other day; the possibility to contact an e-coach any time in the “GoD” condition; and short automated feedback messages after each completed module in the “unguided” condition. Additionally, in order to receive an estimate of the true treatment effect unbiased by assessment dropout, data will be analyzed on ITT basis [100].

Within the framework of our intervention, we will not be able to track the actual amount and frequency of mindfulness practice participants will engage in. Studies have shown that the time spent practicing mindfulness in daily life is an important moderator of intervention efficacy [18]. We will however ask for the average time participants spent practicing at post-assessment and conduct a moderator analysis based on that information. Still, this retrospective assessment might be biased and a future version of StudiCare-M should include immediate tracking of mindfulness practice, e.g., in an accompanying app.

Finally, we will only include college students with moderate to low mindfulness. This approach entails a reduced generalizability of results, not allowing conclusions concerning the efficacy of our IMI for individuals already high on mindfulness. However, we decided to do so because StudiCare-M was designed as an introduction to mindfulness, primarily addressing mindfulness beginners. Consequently, this approach will allow us to evaluate the efficacy of StudiCare-M for those students who need and can therefore benefit from such an intervention the most.

Beside these limitations, our study also offers several strengths.

Many mindfulness studies so far have suffered from samples of limited generalizability. Participants were frequently recruited at only one university or from one study course [30, 34, 101, 102]. Additionally, participants sometimes received money or course credits for study participation [27–29], therefore possibly not representing real students in need. Within the StudiCare project, we are able to overcome these shortcomings. Because participants are mainly recruited via health promotion departments of more than 15 cooperating colleges in Germany, Switzerland, and Austria, we reach students from a great variety of colleges and study courses. Additionally, participants will not receive compensation for their participation reflecting “real life” conditions of health care services (except for a raffle for the long-term follow-up that they will not be informed about until the invitation to the t3 assessment). This approach enables us to learn about real-life demand for and attractiveness of our intervention, important information when it comes to long-term implementation.

So far, only few studies examined the efficacy of IMIs for college students based on acceptance and commitment therapy (ACT) [30, 31, 33, 102]. While mindfulness in general provides useful skills for stress management, ACT goes beyond meditation by emphasizing the concept of psychological flexibility and focusing on values and committed action [59]. These skills might come in specifically handy in a challenging and complex university and subsequent working life. So far, results concerning ACT-IMIs for college students have been promising but have suffered from limitations such as no long-term follow-up [31, 33] or small sample sizes [30, 31]. Our trial is designed to overcome these limitations with a large sample size as well as a 6-month follow-up.

This is one of few studies that examine the effect of “guidance on demand” (GoD) on adherence and efficacy of internet-based interventions [35, 36] and the very first to do so for a mindfulness IMI. As discussed before, mindfulness IMIs could provide an excellent possibility to reach students in need on a population level. Guided IMIs have shown to be most effective but can be associated with increased intervention costs [32]. Consequently, it is worth investigating whether a GoD-IMI for enhancing mindfulness can be effective and well accepted by participants. Additionally, we will compare GoD to an unguided version of StudiCare-M on an exploratory level. Both versions of our intervention are designed according to principles of persuasive design. Therefore, we will gather preliminary evidence on differences between persuasively designed unguided IMIs and IMIs with a minimal form of human guidance concerning effectiveness, adherence, side effects, and adverse events.

While the efficacy of mindfulness IMIs has been shown in many RCTs by now, the questions of how and for whom these interventions work have not been answered sufficiently. Therefore, this RCT will examine a range of different moderators and mediators on an exploratory level in order to contribute to this still understudied area of research [25]. Trials investigating mediators often suffer from methodological limitations such as measuring the proposed mediators, dependent and independent variables only pre- and post-treatment. Consequently, they are unable to establish chronology of change, therefore neglecting a crucial element of investigating mediators [98]. We aim to overcome this shortcoming by conducting an additional assessment at midpoint of the intervention, enabling us to find out at what time changes occur.

One last strength of this RCT is the fact that we will measure potential side effects and adverse events. It has been shown that IMIs can have negative side effects [103–105], but those have been understudied so far [104]. This is especially the case for mindfulness IMIs, where no study so far has investigated such effects. This RCT will therefore contribute to our knowledge on the safety of minimally or unguided mindfulness IMIs, an important precondition for a possible large-scale dissemination of such interventions in the future.

Going to college is associated with high stress levels and an increased risk for the development of mental disorders. Providing effective interventions to college students, helping them improve their coping and stress management skills, can therefore make a valuable contribution to the health and functionality of future society. If effective under the condition of minimal or no guidance, StudiCare-M offers a low-threshold potentially resource-efficient possibility to enhance college student mental health on a population level.

### Trial status

This is protocol version number 1.1 as submitted on 26 July 2019 and amended along reviewers’ comments on 23 October 2020. Recruitment began on 21 May 2018 and was completed by 27 April 2020. Data collection will be completed by November 2020. Prior to recruitment start, the trial was registered at the WHO International Clinical Trials Registry Platform via the German Clinical Studies Trial Register: DRKS00014774 (registration date: 18 May 2018; URL: https://www.drks.de/drks_web/navigate.do?navigationId=trial.HTML&TRIAL_ID=DRKS00014774). In case of important protocol modifications, trial registration will be updated.

## Data Availability

All principal investigators will be given full access to the data sets with the exception of MM. He will be responsible for the blinded analysis of the primary and secondary outcomes and hence will only receive a blinded version of the data needed for this analysis. The data set will be stored on password-protected servers of Ulm University with restricted access. External researchers may get access to the final trial dataset (from HB) on request depending on to be specified data security and data exchange regulation agreements. They can obtain the informed consent form in the same way. To ensure confidentiality, data dispersed to any investigator or researcher will be blinded of any identifying participant information. Anonymized results will be published in peer-reviewed journals and presented on international conferences.

## Abbreviations

ACT: Acceptance and commitment therapy
CBT: Cognitive behavioral therapy
CONSORT: Consolidated Standards of Reporting Trials
GoD: Guidance on demand
IMI: Internet- and mobile-based intervention
ITT: Intention-to-treat
RCT: Randomized controlled trial
StudiCare-M: StudiCare Mindfulness
TAU: Treatment as usual
UG: Unguided
WHO: World Health Organization
WL: Waitlist control group
